# MAGE I Transcription Factors Regulate KAP1 and KRAB Domain Zinc Finger Transcription Factor Mediated Gene Repression

**DOI:** 10.1371/journal.pone.0023747

**Published:** 2011-08-18

**Authors:** Tony Z. Xiao, Neehar Bhatia, Raul Urrutia, Gwen A. Lomberk, Andrew Simpson, B. Jack Longley

**Affiliations:** 1 Department of Dermatology, University of Wisconsin School of Medicine and Public Health, Madison, Wisconsin, United States of America; 2 Department of Molecular Neuroscience, Department of Biochemistry and Molecular Biology, and Gastroenterology Research Unit, Mayo Clinic, Rochester, Minnesota, United States of America; 3 Ludwig Institute for Cancer Research, New York, New York, United States of America; 4 Paul P. Carbone Comprehensive Cancer Center, University of Wisconsin School of Medicine and Public Health, Madison, Wisconsin, United States of America; Kyushu Institute of Technology, Japan

## Abstract

Class I MAGE proteins (MAGE I) are normally expressed only in developing germ cells but are aberrantly expressed in many cancers. They have been shown to promote tumor survival, aggressive growth, and chemoresistance but the underlying mechanisms and MAGE I functions have not been fully elucidated. KRAB domain zinc finger transcription factors (KZNFs) are the largest group of vertebrate transcription factors and regulate neoplastic transformation, tumor suppression, cellular proliferation, and apoptosis. KZNFs bind the KAP1 protein and direct KAP1 to specific DNA sequences where it suppresses gene expression by inducing localized heterochromatin characterized by histone 3 lysine 9 trimethylation (H3me3K9). Discovery that MAGE I proteins also bind to KAP1 prompted us to investigate whether MAGE I can affect KZNF and KAP1 mediated gene regulation. We found that expression of MAGE I proteins, MAGE-A3 or MAGE-C2, relieved repression of a reporter gene by ZNF382, a KZNF with tumor suppressor activity. ChIP of MAGE I (-) HEK293T cells showed KAP1 and H3me3K9 are normally bound to the *ID1* gene, a target of ZNF382, but that binding is greatly reduced in the presence of MAGE I proteins. MAGE I expression relieved KAP1 mediated *ID1* repression, causing increased expression of *ID1* mRNA and *ID1* chromatin relaxation characterized by loss of H3me3K9. MAGE I binding to KAP1 also induced ZNF382 poly-ubiquitination and degradation, consistent with loss of ZNF382 leading to decreased KAP1 binding to *ID1*. In contrast, MAGE I expression caused increased KAP1 binding to *Ki67*, another KAP1 target gene, with increased H3me3K9 and decreased *Ki67* mRNA expression. Since KZNFs are required to direct KAP1 to specific genes, these results show that MAGE I proteins can differentially regulate members of the KZNF family and KAP1 mediated gene repression.

## Introduction

Class I MAGE proteins (MAGE I) are normally expressed only in developing germ cells, trophoblast and placenta because their expression is suppressed in somatic tissues by hypermethylation of promoter region CpG dinucleotide islands [1] [2], [3]. In cancers, however, MAGE I may be aberrantly expressed because of global hypomethylation that often occurs during epigenetic reprogramming [2], [3]. Because MAGE I expression *post utero* is limited to developing sperm and cancers, MAGE I proteins have been used extensively as targets for anti-tumor antibodies and cancer vaccines[4] but the functional contributions of MAGE I to oncogenesis and the mechanisms involved have been relatively understudied.

MAGE I expression in malignancies has been correlated with aggressive clinical course, the acquisition of resistance to chemotherapy, and poor clinical outcome [1], [5], [6], [7], [8], [9]. We have shown that suppression of MAGE I decreases the growth of melanoma and malignant mast cell lines *in vitro* and *in vivo* [10]–[11], and Liu et al [12] have shown that MAGE I expression causes accelerated cell cycle progression, increases the rate of cell migration and invasion *in vitro,* and increases lung metastases in an orthotopic mouse model of human thyroid cancer. We and others have found that MAGE I proteins bind to the RBCC region of KAP1[11], [13], a scaffolding protein whose functions include: **(1).** suppression of p53 by increasing KAP1 ubiquitin E3 ligase activity and p53 ubiquitination [9], [11], [13]. **(2).** global induction and maintenance of transcriptionally inactive heterochromatin characterized by Histone 3 tri-methyl lysine 9 (H3me3K9), and **(3).** suppression of specific genes by localized induction of transcriptionally inactive heterchromatin, targeted by KRAB domain containing zinc finger transcription factors (KZNFs).

The KZNFs are the largest group of transcription factors known in vertebrates, and are involved in cell differentiation, proliferation, apoptosis, tumor suppression, and neoplastic transformation [14], [15] [16]. For instance ZNF382, a KZNF also known as KS1, has recently been identified as a tumor suppressor controlling a number of oncogenes including *ID1* [16]. KZNFs bind to the KAP1 RBCC region through their KRAB domains and to specific DNA sequences through their zinc fingers, thereby guiding KAP1 to repress specific genes by inducing H3me3K9 and causing localized chromatin compaction. Our discovery that MAGE I proteins bind to the same KAP1 RBCC region as KZNFs [11] prompted us to investigate whether MAGE I can affect KZNF and KAP1 mediated gene regulation. Using an integrated reporter gene construct in CHO cells we show here that representatives of two large MAGE I families, MAGE-A3 and MAGE-C2, can de-repress KAP1 mediated gene silencing by ZNF382. In HEK293T cells, MAGE I expression decreases KAP1 binding to the *ID1* oncogene, a ZNF382 target, causing chromatin relaxation with decreased H3me3K9 and increased *ID1* mRNA expression. MAGE I binding to KAP1 also induces ZNF382 poly-ubiquitination and degradation, suggesting the most likely mechanism is degradation of ZNF382, leading to release of KAP1 from the ZNF382 target site. In contrast, MAGE I expression increased KAP1 binding to the *Ki67* gene, causing increased H3me3K9 and decreased *Ki67* mRNA expression. Together, these data indicate that MAGE I proteins can increase expression of some genes while suppressing expression of others. Since KZNFs are required to direct KAP1 to specific sites, this work shows that MAGE I proteins can either positively or negatively regulate KZNF target gene expression, and establish the ability of MAGE I proteins to regulate members of the large and complex KZNF family.

## Results

This work focuses on MAGE-A3 and MAGE-C2 as representatives of two major Class I MAGE families that they are often expressed in human cancers. We will refer to them collectively as “MAGE I”.

### Confirmation of MAGE I and KAP1 binding sites

**Figure 1:**
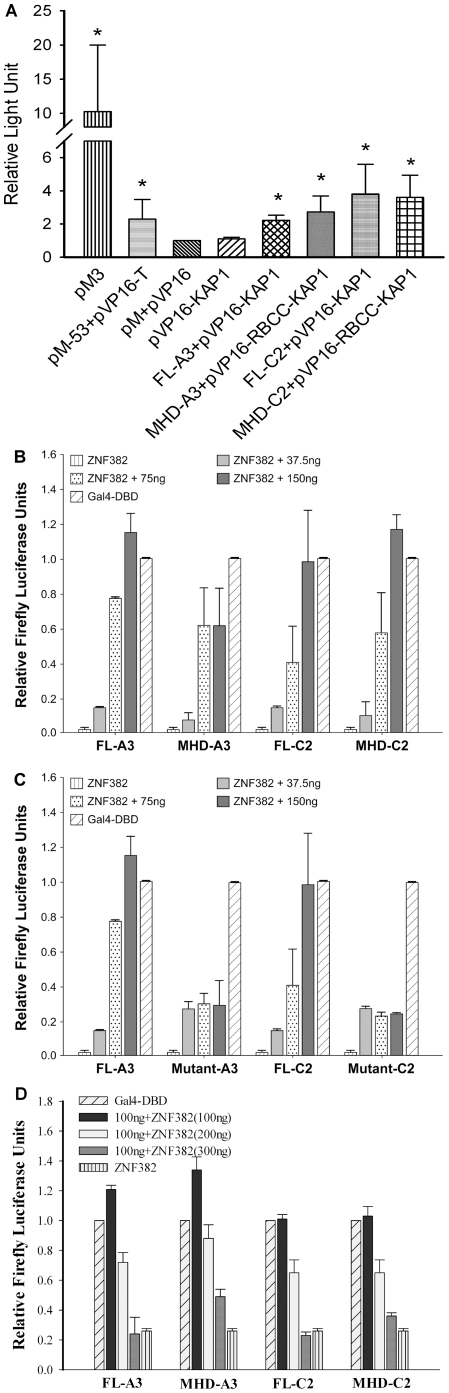
MAGE-KAP1 binding decreases reporter gene repression by ZNF382. A. Full length MAGE-A3, full length MAGE-C2, or corresponding MAGE homology domain constructs, bind to full length KAP1 and RBCC-KAP1 detected by a Mammalian Two-Hybrid Assay. CHO cells transfected with the indicated plasmids were assayed for SEAP (secreted alkaline phosphatase) activity 48 h after transfection, using a chemiluminescent substrate CSPD. pM3, pM53 and pVP16-T are positive control vectors. pM and pVP16 are negative control vectors. pVP16-KAP1 alone shows minimal light units. MHD  =  MAGE homology domain. B. MAGE-A3 and MAGE-C2 decrease KAP1 repression of a ZNF382 responsive reporter gene. Luciferase activities were measured in CHO-5xGal4-UAS-TK-Luc-2p cells transfected with (+) control vector (Gal4-DBD), with vector expressing full length (FL) MAGE-A3 or C2, or with their corresponding MAGE homology domains (MHDs). Maximum repression occurs in the presence of ZNF382 alone. Addition of MAGE expression plasmids causes dose dependent release of repression with increased luciferase activity. C. MAGE-C2 mutant L152A, L153A and MAGE-A3 mutant L120A, L121A, which cannot bind KAP1, fail to relieve repression mediated by ZNF382. D. MAGE-A3 and C2 mediated KAP1 repression of a ZNF382 responsive reporter gene is reversible. Addition of ZNF382 expression plasmids initiate dose dependent regaining of repression with decreased luciferase activity.

MAGE I have a large terminal exon that usually encodes the entire protein and contains the MAGE homology domain (MHD), a conserved region of ∼170 amino acids that comprises about 70% of each MAGE protein and that recently has been implicated as the binding site for KAP1 [2], [13]. To further characterize MAGE-KAP1 interactions we developed constructs of the MAGE-A3 and MAGE-C2 MHDs, and validated their ability to bind to KAP1 in a modified mammalian two hybrid assay [17]. Using this assay, we confirmed our previous findings showing strong binding between full length MAGE-A3 or MAGE-C2 with full length KAP1 (p<0.05) (Figure 1A) [11]. We also found strong binding between the MAGE-A3 and MAGE-C2 MHDs and the KAP1 RBCC region, confirming and extending recently published results [13].

### MAGE I alleviates ZNF382 and KAP1 mediated repression of an integrated reporter gene

ZNF382, also known as KS1, is a KZNF that was recently shown to be a tumor suppressor [16]. To determine the effects of MAGE I expression on ZNF382 function and KAP1 mediated gene repression we used the CHO-5xGal4-UAS-TK-Luc-2p cell line which has limited expression of KAP1 and contains an integrated fusion gene with an optimized binding site for ZNF382 at the 5′ end of a constitutively active luciferase reporter gene [18], [19]. Ectopic expression of ZNF382 recruits KAP1 to the reporter gene and suppresses luciferase expression. Figure 1B shows that co-expression of full length MAGE-A3 or MAGE-C2 relieves ZNF382 mediated repression in a dose dependent manner. Similar results are seen with the corresponding MHDs. We also found that higher levels of expression of ZNF382 expression plasmids lead to dose-dependent recovery of repression with decreased luciferase activity (Figure 1D). Interestingly, mutated MAGE-C2^L152A,L153A^ and MAGE-A3^L120A,L121A^, which have decreased ability to bind KAP1 [13], were unable to relieve ZNF382 induced repression (Figure 1C). Thus, effects of MAGE I on KZNF mediated gene repression require MAGE I binding to KAP1.

### MAGE I attenuates KAP1 binding and repression of *ID1*, a ZNF382 target

**Figure 2:**
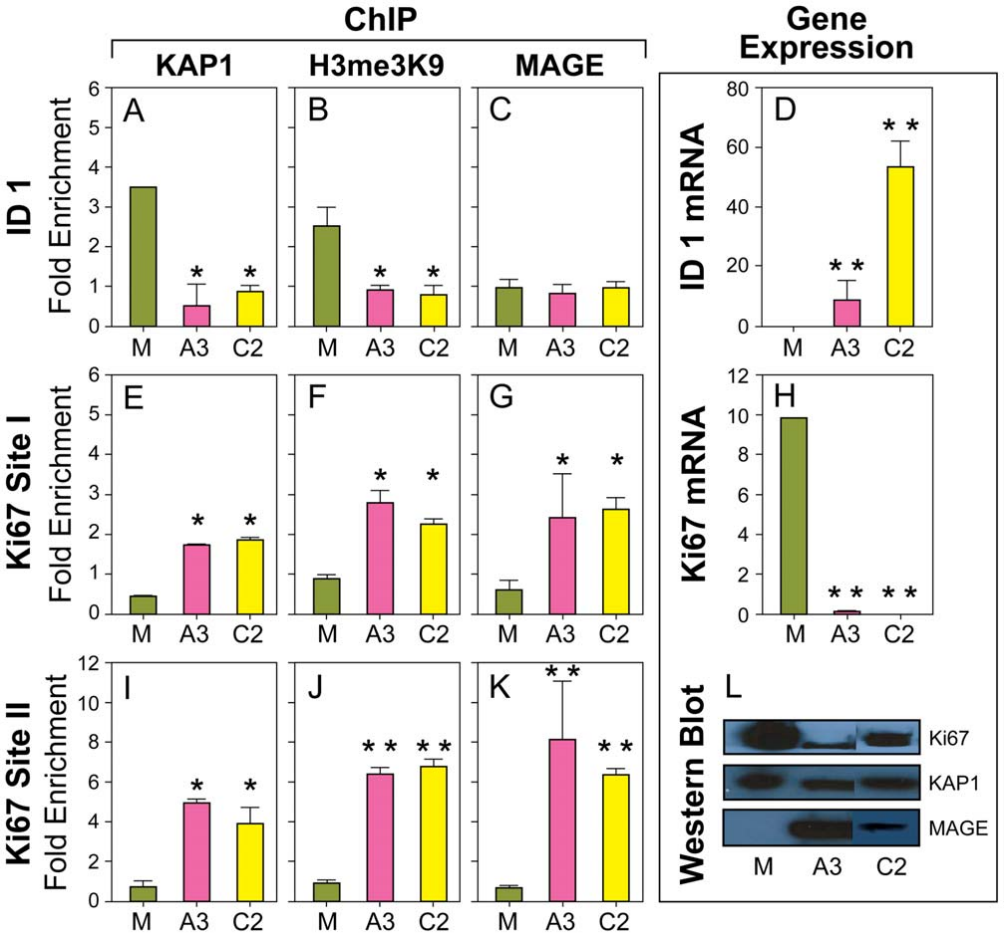
MAGE I regulates KAP1 gene binding, trimethylation of histone 3 on lysine 9, and gene repression in HEK293T cells. MAGE I expression *decreases* binding of KAP1, H3me3K9, and repression of the *ID1* tumor suppressor gene (A, B, D). In contrast, MAGE I expression *increases* binding of KAP1, H3me3K9, and repression of mRNA and protein levels of the *Ki67* gene (E, F, H, I, J, L). Note MAGE I binds to *Ki67* gene sites but not *ID1* gene sites (C, G, K). “M” denotes Mock transfection control. “A3” and “C2” denote MAGE-A3 and MAGE-C2, respectively.

ZNF382 is ubiquitously expressed in normal tissues where it causes localized chromatin compaction and represses several oncogenes including *ID1* [16]. Using ChIP with quantitative real time PCR (qRT-PCR) targeting a unique KAP1 binding site in the *ID1* gene [20], we found that KAP1 is normally bound to the *ID1* gene in MAGE I (-) HEK293T cells and is associated with H3me3K9, indicating KAP1 induced chromatin compaction (Figure. 2A, B). *ID1* mRNA production is low in this state (Figure 2D). As would be expected from our luciferase reporter gene results, expression of MAGE I decreases KAP1 localization to the *ID1* gene (p<0.05), decreases chromatin compaction as measured by H3me3K9 (p<0.05), and increases *ID1* mRNA expression (p<0.01) (Figure. 2A, 2B, 2D). Neither MAGE-A3 nor MAGE-C2 localize to the *ID1* gene (Figure 2C).

### MAGE I enhances KAP1 binding and repression of *Ki67*

To determine whether other KAP1 targets might be affected by MAGE I, we searched for KAP1 binding sites in a genome wide KAP1 data base and found two sites in the *Ki67* gene appropriate for ChIP analysis [20]. We chose to study *Ki67* because *Ki67* is believed to play a role in proliferation and regulation of the cell cycle [21] and we and others have shown that MAGE expression affects the cell cycle [10], [12]. We found that KAP1 occupancy of these sites was very low in control vector transfected cells but increased significantly with expression of MAGE-A3 or MAGE-C2 (p<0.05), indicating MAGE enhances KAP1 mediated binding to *Ki67* (Figure. 2E, 2I). We also found increased H3me3K9 (p<0.05), indicating transcriptionally inactive heterochromatin induced by KAP1 (Figure. 2F, 2J). MAGE-A3 and MAGE-C2 each bound to both KAP1 binding sites, co-localizing with KAP1, as would be predicted from the known ability of MAGE I to bind KAP1(Figure. 2G, 2K) [11], [13] Finally, as we would predict from the increased binding of KAP1 and increased chromatin compaction, *Ki67* mRNA (p<0.01) and protein expression were decreased by MAGE I (Figure. 2H, 2L).

### Mutant MAGE I does not induce KAP1 mediated gene regulation

**Figure 3:**
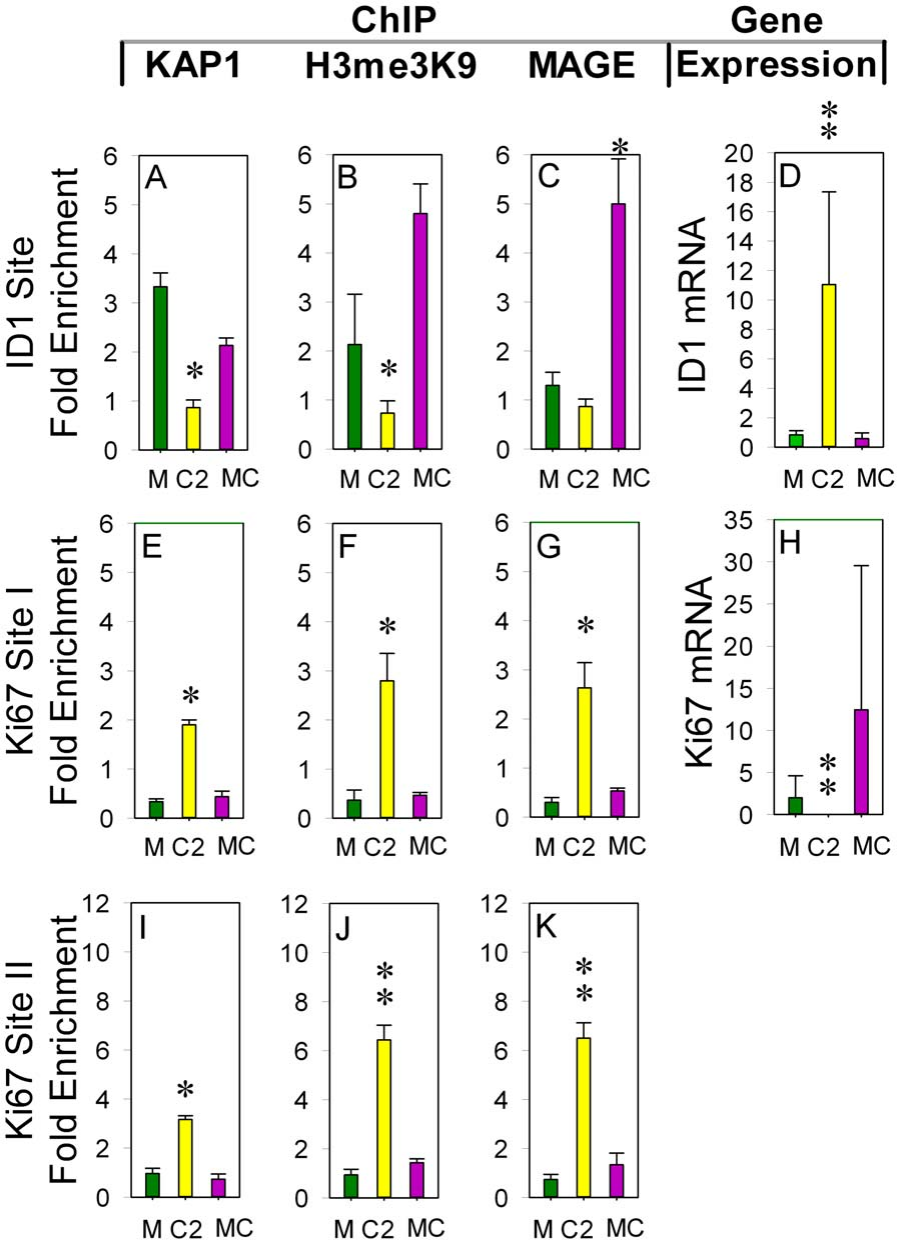
MAGE-C2 mutant fails to regulate KAP1 gene binding, H3me3K9, and gene repression in HEK293T cells. MAGE-C2^L152A L153A^ expression does not affect binding of KAP1, H3me3K9, and repression of the *ID1* tumor suppressor gene (A, B, D). MAGE-C2^L152A L153A^ enrichment is seen at *ID1* sites compared to MAGE-C2 and mock negative control (C). MAGE-C2^L152A L153A^ expression dose not *increases* binding of KAP1, H3me3K9, and repression of mRNA level of the *Ki67* gene (E, F, H, I, J). Note MAGE-C2^L152A L153A^ fails to bind to the *Ki67* gene sites but *ID1* gene sites (C, G, K). “M” denotes Mock transfection control. “C2” denotes MAGE-C2. “MC” represents MAGE-C2^L152A L153A^.

**Figure 4:**
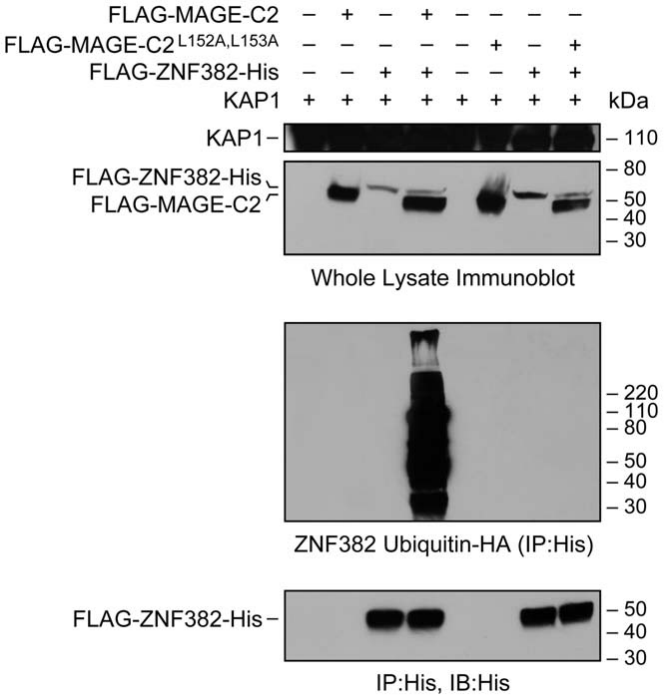
MAGE–KAP1 binding induces ZNF382 ubiquitination in HEK293T cells. HEK293T cells were transiently transfected with His tagged wild-type MAGE-C2 (WT), mutant (non-binding) MAGE-C2^L152A L153A^ , or empty vectors (Mock) and co-transfected with ZNF382, KAP1, and HA tagged ubiquitin. Cells were incubated for 5 hours in the presence of 25 µM MG132 (C2211, SIGMA). Top two panels: ZNF382 (around 55 kDa), MAGE-C2 (around 50 KD) and KAP1 (110 KD) were detected in whole lysates by immunoblotting with anti-KAP1 (upper panel) and anti-FLAG antibodies (lower panel). Third panel: His tagged ubiquitinated proteins were immunoprecipitated with anti-His and detected with anti-HA. High-molecular-weight ubiquitinated species were seen only in blots of cells transfected with both ZNF382 and MAGE-C2. Note that no ubiquitination occurred when wild type MAGE-C2 was replaced with MAGE-C2^L152A L153A^ which does not bind to KAP1, indicating MAGE–KAP1 binding is required for ZNF382 ubiquitination. Also please note an ubiquitinated ZNF382 degradation product of lesser molecular weight in the presence of wild type MAGE-C2 and KAP1. Lowest panel: immunoprecipitation and immunoblotting confirms expression of ZNF382.

To determine whether MAGE I mediated gene regulation requires MAGE I - KAP1 binding, we repeated ChIP and mRNA expression experiments with ectopic over-expression of mutant MAGE-C2^L152A,L153A^, which has decreased ability to bind KAP1, and specifically abrogates MAGE I mediated substrate ubiquitination by KAP1 E3 ligase [13]. We found that KAP1 remains bound to the *ID1* gene in HEK293T cells expressing ectopic MAGE-C2^L152A,L153A^ and is tightly associated with H3me3K9 compared to cells with wild type MAGE-C2 (Figure. 3A, 3B). Consistent with these findings, we did not see an increase of *ID1* mRNA (Figure. 3D). Interestingly, although it does not induce ZNF382 ubiquitination, MAGE-C2^L152A,L153A^ is still somewhat localized to the *ID1* gene (Figure 3C) raising the possibility that it retains residual capacity to bind KAP1 or another protein in the KAP1/chromatin complex (Figure 4). This result supports the hypothesis that MAGE I specifically mediates de-repression by altering KZNF ubiquitination and degradation. MAGE-C2^L152A,L153A^ fails to enhance KAP1 and H3me3K9 binding to *Ki67* gene (Figure. 3E, I, F, J), and MAGE-C2^L152A,L153A^ does not bind to *Ki67* gene (Figure. 3G, 3K). Predictably, *Ki67* mRNA expression is not decreased by expression of MAGE-C2^L152A,L153A^ (Figure 3H). Overall, these results show that proper binding of MAGE I to KAP1 is required for MAGE l mediated gene regulation and implicate ubiquitination in the mechanism, but leave open the possibility that MAGE I may have other interactions with KAP1-chromatin complexes.

### MAGE-C2 enhances-ZNF382 ubiquitination

It is known that MAGE I enhances KAP1 ubiquitin ligase activity and specifically increases ubiquitination and degradation of p53 [13]. To determine whether MAGE could alter KAP1 localization by increasing ubiquitination and degredation of a KZNF, we investigated ZNF382 ubiquitination in the presence of wild type and mutant MAGE-C2. MAGE-C2 expression enhanced ZNF382 ubiquitination and degradation in an *in vivo* ubiquitination reaction (Figure 4). ZNF382 poly-ubiquitination requires MAGE-C2 binding to KAP1, since non-binding mutant MAGE-C2^L152A,L153 A^ does not increase ZNF382 ubiquitination. Because binding to KAP1 is necessary for KZNF suppression of specific genes, these results suggest that MAGE-C2 may prevent significant repression of a KZNF target gene by increasing ubiquitination and degradation of KZNFs that bind to that gene.

## Discussion

The nearly tumor specific expression patterns of MAGE I proteins make them ideal therapeutic targets, and clinical and *in vitro* evidence is accumulating that MAGE I expression contributes to cancer chemoresistance and growth, cell survival, and metastasis [1], [5], [6], [7], [8], [9]. We and others have previously shown that common functions of MAGE are to bind to KAP1 and suppress p53 [9], [10], [13] but the biochemical functions of MAGE I proteins remain incompletely understood. In the present work we demonstrate that MAGE I expression can regulate ZNF382, an important KZNF tumor suppressor, and can affect the ability of ZNF382 and KAP1 to bind and regulate a downstream target, the *ID1* oncogene. MAGE expression suppresses ZNF382 function, decreasing KAP1 binding to *ID1* and promoting *ID1* expression, in line with previously observed pro-oncogenic effects of MAGE I. We also show MAGE I can affect KAP1 binding to the *Ki67* gene, with seemingly opposite effects, including increased KAP1 binding, increased chromatin compaction, and decreased gene expression. Additionally, MAGE-C2^L152A,L153A^ fails to regulate gene expression, indicating that proper binding between MAGE I and KAP1 is required for MAGE I regulation of gene expression.

ZNF382 is a tumor suppressor that is ubiquitously expressed in normal tissues, where it recruits KAP1 and causes chromatin compaction and suppression of several oncogenes including *ID1* [16]. Expression of ZNF382 has been reported to be lost in cancers due to gene deletion or hypermethylation, but ZNF382 expression and tumor suppression are expected in hypomethylated states [16]. Hypomethylated states support MAGE I expression, and our data show unequivocally that MAGE I can suppress ZNF382 function, thereby relieving repression of ZNF382 downstream targets and inducing oncogene expression. Thus, MAGE I expression offers a mechanism for ZNF382 suppression and oncogene activation in the absence of DNA hypermethylation or ZNF382 gene deletion.

It has been shown that MAGE I enhances E3 ubiquitination of p53 by increasing KAP1 ubiquitin E3 ligase activity through recruitment and/or stabilization of E3 ubiquitin-conjugating cascades [13]. Our data show that MAGE I induces ubiquitination and degradation of ZNF382 and that binding of MAGE to KAP1 is required, suggesting that MAGE I can also increase KAP1 ubiquitination of KZNFs. However, other possible mechanisms such as ubiquitin receptor modification or interference with the assembly of the basal transcription apparatus by the ubiquitin moiety itself cannot be completely excluded [13], [22]. While it appears clear that MAGE I can decrease KAP1 binding in the case of the ID1 gene, probably by increasing ubiquitination and degradation of KZNF382, there are at least two other potential mechanisms by which MAGE I could affect KAP1 localization and gene repression. Since both MAGE I and KZNF proteins bind to the KAP1 RBCC region, overlap or close proximity of the KAP1 motifs recognized by MAGE I and KZNFs could result in competition, with decreased KZNF binding to KAP1 leading to decreased KAP1 recruitment to specific sites. Alternatively, MAGE I expression may stabilize binding between KZNFs and KAP1 without increasing ubiquitination, a situation which fits the data observed for *Ki67* where we see increased binding of KAP1 associated with MAGE I binding to the same site, presumably mediated by an unknown KZNF. The fact that MAGE I protein co-localizes with KAP1 to *Ki67* supports this model.

It is important to note that the effects of MAGE-A3 and MAGE-C2 are thus far always similar for a given gene and do not appear to be random, indicating a true biologic effect. Since both MAGE-A3 and MAGE-C2 have similar effects on each of the genes studied (*i.e.,* decreased KAP1 binding of *ID1* and increased KAP1 binding to *Ki67*) the reason for the opposite effects does not appear likely to reside in the MAGE I proteins and is more likely a function of the particular KZNF. KZNFs bind KAP1 by their KRAB domains, which are heterogeneous and may contain members of one or both of several KRAB A and KRAB B motifs [23]. We speculate that different KRAB domains may bind to KAP1 with differing affinities or at slightly different sites, or may interact with the ubiquitin ligase complexes in different ways, leading either to increased KZNF ubiquitination and decreased binding seen with *ID1*, or to increased binding and co-localization with MAGE I seen with *Ki67*. Except for sites of DNA damage, where KAP1 binds DNA in a sequence independent fashion, KAP1 normally requires binding to KZNFs to guide it to specific genes. KAP1 binds to ∼7,000 sites in the human genome [20] and there are about 270 KZNFs, indicating that some KZNFs must bind to more than one site. Unfortunately, antibodies are not available for most KZNFs and it is not known where most of them bind, making it problematic and beyond the scope of this work to completely analyze their regulation and function, and thus identify the KZNFs that bind to *Ki67*. While the results with *Ki67* seem to indicate an anti- proliferative affect of MAGE I proteins, it is important to consider the role of MAGE I in the DNA damage response, which requires a temporary pause in the cell cycle for DNA repair. Interestingly, when looked at over the course of 72 h we saw an initial drop in Ki67 expression followed by slow partial recovery, compatible with a temporary cell cycle halt (data not shown). While it remains to be determined how MAGE I regulation of *Ki67* may affect overall cell proliferation or DNA damage repair, when these data are combined with our studies of ZNF382 they show unequivocally that MAGE I proteins may have differential effects (repression or de-repression) on different KAP1 KZNF targets.

**Figure 5:**
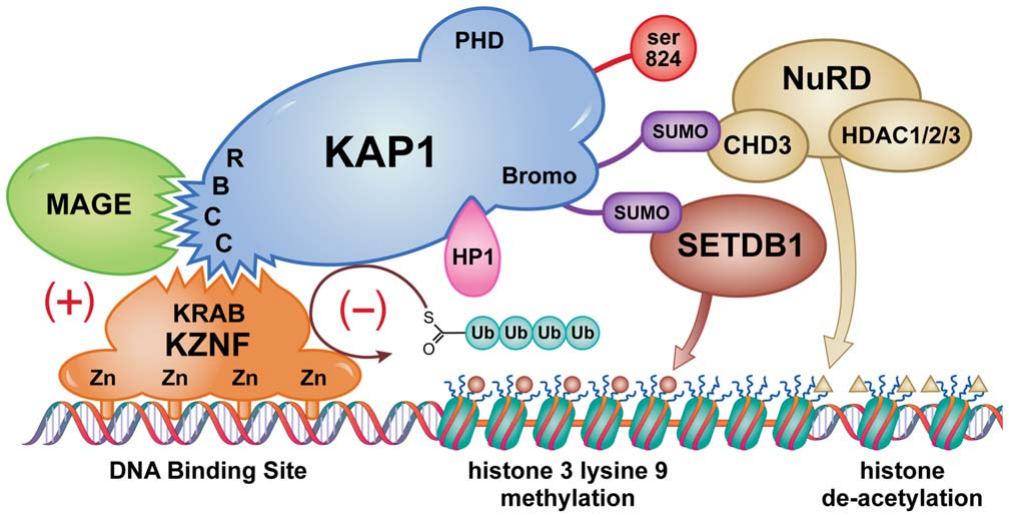
Proposed model of MAGE-KAP1 interactions. KAP1 performs diverse functions by serving as a molecular scaffold that binds multiple proteins which allow it to regulate chromatin environments. KAP1 has an N terminal RING-B-box coiled-coil (RBCC) domain that binds to the KRAB domains of KZNFs, which target KAP1 to specific DNA sequences through their zinc finger DNA binding motifs. KAP1 mediates localized compaction of euchromatin to heterochromatin that is necessary for suppression of specific gene transcription, and that is associated with chromatin modifications including histone de-acetylation, histone 3 tri-methylation on K9, and HP1 binding to both DNA and histones. In some cases, MAGE expression enhances KAP1 E3 ubiquitin ligase activity, resulting in KZNF ubiquitination and degradation, thereby de-repressing KZNF mediated gene repression, shown as (-). In other cases, MAGE enhances KZNF and KAP1 localization to specific gene loci, shown as (+) (After A. Ivanov [22]).

Because transmission of signals in biologic systems may be complex and intertwined, it is often problematic to assign a direct causal relationship to regulation of specific genes by an individual molecule or sets of molecules. Genes in areas of chromatin relaxation may still be negatively regulated by other mechanisms, so removal of KAP1 mediated repression by MAGE I expression does not guarantee protein expression. However, the ability of KZNF targeted KAP1 to suppress specific genes by causing localized chromatin condensation trumps other forms of regulation because if the gene is condensed, it cannot be transcribed. Therefore, our finding that MAGE I expression affects KAP1 binding and chromatin condensation unequivocally defines direct gene regulation by MAGE I proteins (Figure 5).

## Materials and Methods

### Tissue culture and transfections

HEK293T were purchased from American Type Culture Collection and were cultured in DMEM with 10% fetal bovine serum and 1% antibiotics (penicillin, streptomycin). CHO-5xGal4-UAS-TK-Luc-2p cells were cultured in F12 with 10% fetal bovine serum and 100 ug/ml hygromycin antibiotic [18], [19]. For transfections with MAGE-A3 or C2, we used calcium phosphate (Invitrogen) and Lipofectamine 2000 (Invitrogen) according to the manufacturer's recommendations.

### RNA isolation, reverse transcription and qRT-PCR

RNA from cells was isolated using a total RNA isolation mini kit (Qiagen). Total RNA was treated with 2 units of DNase I (New England Biolabs). Reverse transcription and qRT-PCR were performed using an Advantage RT-for-PCR kit (Clontech) and SYBR Green Core PCR reagents (SABiosciences), according to manufacturer's recommendations. Quantification of qRT-PCR was done using an ABI Prism 7000 machine and ABI Prism 7000 SDS software (Applied Biosystem). Sequences of primers used for qRT-PCR are presented in Table 1.

**Table 1:** Real time PCR primers for target gene screening.

Primer name	Sequence (5′ → 3′)	Size
KAP1 Binding on Ki67-1F	CTCCGTCTGTGGCACGGGAA	108 bp
KAP1 Binding on Ki67-1R	TGCCGGCACTGTACCGAGC	
KAP1 Binding on Ki67-2F	CCCTGCCTGACAGGAACACGC	70 bp
KAP1 Binding on Ki67-2R	GCGCTACTGTGGAGCTGGGG	
KAP1 Binding on ID1-1F	TGCAGGTTTGGCCTATGCTGAG	103 bp
KAP1 Binding on ID1-1R	AAAGGGCCATTGCTGCCCAGG	
Ki67-1F	TTGGAGAATGACTCGTGAGC	218 bp
Ki67-1R	GGAAGCTTTCAATGACAGGA	
ID1-1F	TGGAGATTCTCCAGCACGTC	181 bp
ID1-1R	ATGCGATCGTCCGCAGGAAC	
GAPDH-1F	GAGTCAACGGATTTGGTCGT	238 bp
GAPDH-1R	TTGATTTTGGAGGGATCTCG	

### Protein isolation

To obtain the whole-cell lysate for western blot analysis, cells were lysed using denaturing RIPA buffer containing phosphate buffer saline (pH 7.4), 0.5% sodium deoxycholate, 0.1% SDS, 1% (v/v) NP-40, 100 mM sodium orthovanadate, and proteinase inhibitor cocktail (Sigma).

### Antibodies

For human target validation studies and immunoprecipitation we used: anti–MAGE-C2 (polyclonal, Santa Cruz Biotechnology), anti-human KAP1 (polyclonal, Novus Biologicals, recognizing the NH_2_-terminal region 1–50 amino acids of KAP1), anti-KAP1 monoclonal 20C1 (ChIP grade, Abcam), anti-FLAG monoclonal M2 (Sigma) and anti-human H3me3K9 (monoclonal, E6204, SABiosciences), and anti-human *Ki67* (polyclonal, ab15580, Abcam).

### Expression vectors

FLAG-tagged wild type or mutant MAGE-A3 or C2 were expressed using p3xFLAG-CMV-2 plasmid (Sigma). ZNF382 with His tag at the 3′ end was also used in this vector.

### Site-Directed Mutagenesis

Site-directed mutagenesis was done by overlap extension PCR (Invitrogen). MAGE-C2 mutation: L152A, L153 A. The primers including 50 forward ( GGT GGC CGA GTT AGT GGA GTT CGC AGC ACT CAA ATA CGA AGC AGA GGA GC) and 50 reverse (GCT CCT CTG CTT CGT ATT TGA GTG CTG CGA ACT CCA CTA ACT CGG CCA CC) were used to allow substitutions L152A and L153A in the MHD region of MAGE-C2. MAGE-A3- L120A, L121A was introduced using the primers 50 forward (GGT GGC CGA GTT GGT TCA TTT TGC AGC ACT CAA GTA TCG AGC CAG GGA GC) and 50 reverse (GCT CCC TGG CTC GAT ACT TGA GTG CTG CAA AAT GAA CCA ACT CGG CCA CC). PCR conditions included one cycle at 94°C for five minutes and 30 cycles of: 94°C for 30 sec, 60°C for 30 sec and 72°C for 30 sec, followed by one cycle of 72°C for seven minutes. PCR products were run on an agarose gel to confirm the presence of a product of correct molecular weight. Sequencing was performed to confirm the mutagenesis.

### Mammalian Two-Hybrid assay

Mammalian two hybrid studies were performed after modification of the Matchmaker™ system (Clontech Laboratories Inc), essentially according to the instructions of the manufacturer, with SEAP secreted reporter protein. The modified bait expression plasmid contains an SV40 promoter driving expression of a hybrid of full length (FL) or MHD MAGE-A3 or C2 and a DNA-binding domain (DBD). The modified prey expression plasmid uses the SV40 promoter driving a hybrid protein of an activation domain (AD) and FL or RBCC region KAP1. Binding of the bait and prey plasmids cis-activates E1b which in turn transactivates a reporter plasmid containing a secreted alkaline phosphatase (SEAP) cDNA. Phosphatase produced by the transactivated SEAP plasmid is measured by enzyme-based detection using the chemiluminescent substrate CSPD.

### Chromatin immunoprecipitation

ChIP assays were performed according to the protocol of the kit manufacturer (SABiosciences). Equal amounts of specific and nonspecific control IgG were used. Both input DNA and immunoprecipitated DNA were purified with SABiosciences DNA mini kit. All samples were analyzed by qRT-PCR and were carried out in triplicate with primers specific for these regions using SYBR Green Supermix (SABiosciences). The fold enrichment of target sequence was determined using the following formula: fold enrichment  = 2^(ΔCT of input − ΔCT of IP'ed DNA)^.

### Luciferase reporter assay

To test how MAGE I affects ZNF382, CHO cells with a ZNF382 luciferase reporter 5xGal4-UAS-TK-Luc-2p were evaluated after transfected with ZNF382 with or without MAGE-A3 or C2 or mutants. Luciferase assay was done as previously described [18], [19]. All the experiments were performed in triplicates.

### In vivo ubiquitination assay

8 µg of wild-type or mutant MAGE-C2 or mutant expression plasmids, or empty vector control, were introduced into HEK293T cells at 70% confluency using the CaPO4 method (Clotech). 8 µg FLAG-ZNF382-His tag, 4 µg KAP1, 4 µg of pcDNA 3.1+ vector harboring HA tagged ubiquitin were co-transfected. After 20 h, the cells were treated with 25 µM of MG132 (C2211, Sigma) for 5 h, lysed in RIPA buffer, and subjected to immunoprecipitation. A 500 µg aliquot of total protein from the transfected HEK293T cells in 250 µl lysis solution was mixed with 10 µl of the anti-His affinity matrix (Cell signaling) pre-blocked with 2% bovine serum albumin (BSA), and incubated overnight with gentle rotation at 4°C. The affinity matrix was washed with HIPA buffer three times, collected by centrifugation, and the precipitated proteins were denatured in sample buffer containing 0.1 M dithiothreitol (DTT), subjected to 7% SDS-PAGE and transferred to a PVDF membrane. The transferred proteins were then incubated with anti-His (high affinity HA 3F10, Cell signaling), or anti-HA (monoclonal, Cell signaling) antibodies. Anti-mouse IgG, HRP-linked secondary antibodies (Cell signaling) were used for the western blotting procedure.
